# Does the KDEL receptor cycle between the Golgi and the ER?

**DOI:** 10.1038/s41467-024-45849-0

**Published:** 2024-03-20

**Authors:** Fernando Aniento, David G. Robinson

**Affiliations:** 1 Departamento de Bioquímica y Biología Molecular, Facultad de Farmacia, Instituto Universitario de Biotecnología y Biomedicina (BIOTECMED), Universitat de València, 46100 Burjassot, Spain; 2 Centre for Organismal Studies, Univ. Heidelberg, 69120 Heidelberg, Germany

**Keywords:** Cell biology, Membrane trafficking

**arising from** J.C. Alvim et al. *Nature Communications* 10.1038/s41467-023-37056-0 (2023)

KDEL receptors (KDELRs) are ubiquitous seven-transmembrane domain proteins. In the early Golgi, they bind to endoplasmic reticulum (ER)-resident proteins with a C-terminal Lys-Asp-Glu-Leu (KDEL) sequence and facilitate their retrieval back to the ER. Recognition of ER proteins by KDELRs is pH dependent with binding occurring at low pH in the Golgi and release under conditions of higher pH in the ER. Crystallographic data has shown that ligand binding to KDELR results in the movement of transmembrane domains 1 and 6. This causes the formation of an H-bond that locks the KDEL-ligand in the receptor and activates the KDELR for COPI binding and subsequent COPI vesicle formation^1,2^. KDELR cycling via COPII and COPI vesicles is a scenario presented in all major cell biology textbooks, with the molecular mechanisms being described in detail in a recent review^3^. Despite being universally accepted by animal cell biologists the KDELR cycling scenario has now been challenged^4^ in a paper recently published in *Nature Communications*.

This paper is based in a previous one where these authors measured the ability of the K/HDEL receptor (ERD2) to retain the neutral cargo molecule barley α-amylase (AMY) tagged with HDEL (AMY-HDEL)^5^. Using this assay, they showed that, unlike ERD2 itself, C-terminal fluorescently tagged ERD2 constructs were unable to reduce AMY-HDEL secretion and thus concluded that C-terminal ERD2-(X)FP fusions are non-functional. This contradicts data obtained from similar experiments previously performed by Montesinos et al. ^6^ who used protein immunoblotting to monitor the secretion of a bona fide endogenous HDEL-ligand, the chaperone BiP. While ERD2a-YFP had no effect on the secretion of Sec-GFP, BiP secretion was strongly inhibited in the presence of ERD2a-YFP. It is also in disagreement with earlier data obtained on animal cells. For example, Lewis and Pelham^7^ used a human KDELR with a C-terminal myc tag to show a ligand-induced redistribution of the KDELR. This effect was not observed in a mutant with altered ligand specificity, indicating that this was not a consequence of the c-myc tag, but rather the result of ligand binding^7^. In this regard, it should also be noted that later studies have shown that myc-FLAG-tagged KDEL receptors remain functional and are able to retrieve both endogenous KDEL proteins from the Golgi as well as exogenous reporter proteins^8,9^. Another example is that of Majoul et al.^10,11^ who used a human KDELR C-terminally tagged with CFP/YFP and showed that binding of a KDEL ligand (cholera toxin A subunit) nevertheless induced KDELR homo-oligomerization. Ligand binding to the C-tagged KDELR also increased its interaction with components of the molecular machinery involved in COPI vesicle formation (ARF1, p24 proteins and COPI subunits). Indeed, KDEL receptors and p24 proteins are among the top hits of the core proteome of mammalian COPI vesicles^12^. Arabidopsis ERD2a-YFP has also been shown to interact with p24 proteins, ARF1 and COPI subunits^6^.

As an alternative strategy for visualizing KDELR trafficking, Silva-Alvim et al.^4,5^ have generated a novel fluorescent ERD2b construct: an N-terminal fluorophore followed by an extra transmembrane domain followed by ERD2b itself. Interestingly, (X)FP-TM-ERD2 can bind HDEL ligands but is exclusively Golgi-localized and did not show any redistribution to the ER even in the presence of HDEL ligands. Using this construct, they also claim that this exclusive Golgi residency is conserved in eukaryotes. This unexpected result raises questions as to the fidelity of this construct in representing KDELR trafficking. In contrast to KDLERs (including ERD2a, b) the (X)FP-TM-ERD2b construct of Silva-Alvim et al.^3,4^ has 8 rather than 7 transmembrane domains and is based on the structure of so-called ERPs (ERD2-related proteins), which do not seem to play a role in ER retention of soluble proteins. Silva-Alvim et al.^4^ have shown that mutation of lysine residues in the C-terminus of their ERD2 construct does not alter its biological activity (ligand binding). Consequently, they claim that the model proposing that KDEL ligand binding induces a conformational change exposing these lysine residues to form a retrieval motif involved in COPI-dependent Golgi-ER transport^1^ is incorrect. However, mutation of the equivalent lysine residues in a chicken ERD2 version without any extra TM domain caused this mutant to remain Golgi localized upon KDEL ligand overexpression^1^. Moreover, KDELR mutants unable to bind KDEL ligands also remain Golgi localized upon KDEL ligand overexpression, linking ligand binding to KDELR transport^1^. The predicted conformational change for the chicken KDELR upon ligand binding requires rearrangement of transmembrane (TM) domains 1 and 6^1^ and thus it is difficult to predict whether the addition of an extra TM domain before TM1 can interfere with this conformational change. It also remains unclear whether other essential features of KDELR trafficking are unaffected, e.g., oligomerization (which has been shown for mammalian^10,13^ and plant^14^ ERD2 proteins), or the interaction with p24 proteins, ARF1 and COPI subunits (which has been shown for mammalian^10^ and plant^6^ ERD2 proteins.

ERD2-mediated recruitment of ARF-GAP^13^ which may cause dissociation of COPI from the Golgi, has been used by Silva-Alvim et al.^5^ as an argument against the recycling function of ERD2. However, there are a number of reports showing a role of ARF-GAP in cargo recruitment and COPI vesicle formation, although the exact timing of GTP hydrolysis on ARF1 still needs to be established. The fact that several cargo proteins (including SNAREs or the KDEL receptor) bind ARF-GAPs, suggests that, in addition to their canonical role in GTP hydrolysis, ARF-GAPs may also function as cargo adaptors to sort proteins within COPI vesicles^15^.

It is unfortunate that Silva-Alvim et al.^4,5^ fail to cite several recent papers on animal cells that clearly demonstrate that endogenous KDELR redistributes from the Golgi to the ER upon ligand binding. For example, Giannotta et al.^16^ and Cancino et al.^17^ both used KDEL-BIODIPY633, that can permeate membranes and binds to KDELR to follow the retrograde transport of the receptor after exposure to KDEL ligands. In addition, Cancino et al.^17^ used a KDELRVSVG chimera, which like KDELR cycles between the ER and the Golgi at 32 °C but unfolds at 40 °C and becomes trapped in the ER. Even more convincing are the data of Gerondopoulos et al.^18^, who used mScarlet tagged KDEL ligands, which also enable a Golgi-to-ER redistribution of KDELR to be visualized. As controls they employed retrieval signals having a much lower affinity than KDEL as a ligand (ADEL, DDEL). In contrast to KDEL, the latter do not lead to a retrograde transport of KDELR, again linking ligand binding to KDELR transport. To avoid possible interference with sorting signals for COPI binding Bräuer et al.^1^ and Gerondopoulos et al. ^18^. followed the trafficking of KDELR using a KDELR construct with a 20 aa spacer between the receptor and the fluorescent tag.

Some of the points related to the fidelity of (X)FP-TM-ERD2b as a reliable marker for monitoring EDR2 dynamics have previously been voiced^19^, but strangely Alvim et al.^5^ did not address these criticisms in their recent paper. We maintain that the evidence in favor of this “classical” scenario is overwhelming, certainly so for animal cells, being based on solid experimental evidence provided by several independent laboratories. In contrast, the support for the alternative model mostly relies on an artificial 8 transmembrane domain protein that is different from the “real” endogenous receptor and still needs to be characterized. For example: is it able to change conformation upon ligand binding? Can it oligomerize (as it is the case of mammalian or plant ERD2 proteins)? Can it interact with p24 proteins, ARF1 or COPI subunits? In the absence of such data we believe that it is premature to rule out retrograde trafficking of KDELRs from the Golgi to the ER in response to the presence of K/HDEL ligands.

However, as previously pointed out by us *“A common feature of the steady state distribution of KDELR in mammals and ERD2 in plant cells is that receptor labeling of the ER is very low. Only when the receptor or HDEL-ligands are overexpressed does KDELR/ERD2 diffuse out into the body of the tubular ER system”*^19^. The problem of KDELR detection in the ER in plant cells is exacerbated due to the unique structural features of the early secretory pathway in (higher) plant cells^20^. A recycling compartment like the ERGIC (ER-Golgi intermediate compartment) of mammalian cells does not exist in plants, its functions being assumed by the *cis*-most Golgi cisternae. However, since Golgi stacks lie closely appressed to the surface of the ER, it may be difficult to differentiate between Golgi-localized KDELR and KDELR transitorily localized in the ER giving the impression that KDELR in plants does not exit the Golgi. On the other hand, cycling of KDELR between the ER and Golgi has been shown for yeast^21^ which not only lacks an ERGIC, but also does not have stacked Golgi cisternae^22^. Nevertheless, and despite varying Golgi-ER morphologies, common to all three organismal types is the operation of a vesicular cycling machinery between the ER and Golgi (using COPII coats for anterograde, and COPI coats for retrograde traffic). Moreover, since KDEL receptors appear to be ubiquitous in all eukaryotic organisms, we see no a priori reason for assuming that a KDELR-based ER-retrieval system should also not be operable in plants.
